# Northampton General Hospital

**Published:** 1905-07-22

**Authors:** 


### HOSPITAL ADMINISTRATION.

### CONSTRUCTION AND ECONOMICS.

### NORTHAMPTON GENERAL HOSPITAL.

THE NEW HOSPITAL COMPLETED.

A dinner was given at the Grand Hotel, Northampton, on the 12th inst. by Mr. F. G. Adnitt, J.P. (Chairman of the General Building Committee), and Mr. John Cooper, J.P., the Vice-Chairman, to the members of the Building Committee and the Honorary Medical and Surgical Staff for the purpose of celebrating the completion of the new buildings of the Northampton General Hospital. Mr. F. G. Adnitt, J.P., presided, and was supported by Sir Henry Burdett, who is responsible for the inception of the rebuilding, and has superintended the work throughout, the Mayor (Councillor A. E. Marlow), the Ex-Mayor (Councillor E. Lewis), and Mr. Bruce Cooper (representing his father, Mr. John Cooper, J.P., who was absent through indifferent health). There were also present the Rev. T. C. Beasley, Councillor H. Martin, J.P., Mr. H. Manfield, J.P., Mr. F. W. Dorman, Dr. R. A. Milligan, Dr. P. S. Hichens, Mr. G. H. Percival, Dr. Harries Jones, Mr. W. R. D. Adkins, J.P., C.C., Mr. J. Rushton, Mr. C. A. Markham, Assistant Commissioner Woolston, Mr. R. Cosford, Mr. C. S. Risbee, Mr. F. W. Smith, etc. Apologies were received from Earl Spencer, K.G., the Marquis of Northampton, Lord Lilford, Sir Charles Knightley, Bart., Mr. John Cooper, J.P., Mr. R. B. Loder, Dr. Richard Greene, Dr. Buszard, Mr. T. P. Dorman, Mr. A. Page, J.P., Mr. W. Smith, Mr. S. P. Bennett, etc.

The Chairman, in giving the loyal toasts, referred to the great interest the King took in hospital work. He expressed his regret that Dr. Buszard, who had done so much for the Northampton Hospital, could not be present, and also Mr. John Cooper. He also extended a hearty welcome to Sir Henry Burdett, and said they recognised the worth of his work for the Northampton Hospital, especially in view of the fact that he was an undoubted expert in all departments of hospital work.

Sir Henry Burdett said it was a great pleasure to him to be there that night, because he was in the position of one who could look backward aud look forward. Looking backward, he remembered the problem he was set when he was called in by the governors of the Northampton General Hospital to determine what steps should be taken to make the old building what all hospitals should be, a guest-house for the sick. He found the state of affairs to be incidental to the use of buildings extending over a century, where but few structural alterations had taken place, and modern hygiene was practically ignored. The problem which had to be solved was whether it was possible to re-construct. There were circumstances which would have made that possible of accomplishment, but there were others which would have made it exceedingly inadvisable. Under the old methods of hospital treatment before the true meaning of dirt and of the diseases resulting from it were fully realised it was considered sufficient if the annual cleaning of such an establishment meant simply the recolouring of the walls. Such a procedure made the place look smart, but its result was to carefully husband all the impurities, and to bind them together for a long period of years. Thus in wards where the hygienic breakdown forced the governors to seriously face the position he found he could with his umbrella penetrate the wall to a depth of nearly two inches, the colour-wash covering an accumulation of filth. In addition the system of ventilation was very defective. He had a quantity of the impurities in the ventilators collected together with a portion of the colour-wash. Under the floor he discovered what was, in a hygienic sense, dangerous filth equal in volume to three tons weight. Dr. Sims Woodhead spent some three months in cultivating the various products discovered. The speaker had photographs of the cultures of the various and variegated specimens of filth taken from the old infirmary, and he thought that some day, in order to show the wisdom of those men who had the courage to vote for the scheme, whose successful accomplishment they were celebrating, he would illustrate on a screen the diseases which the poorer inhabitants of Northampton had escaped through the erection of the new institution. Although he presented a report which gave the governors the alternative of reconstructing, he found when making explorations with a view to increasing the number of windows that there was a possibility of the collapse of one side of the roof, and the result was that they wisely decided to accept the other alternative, and build a new hospital apart from the old building, and to convert the latter into an administration block.

Sir Henry said he had spent the whole day in the new hospital and administration block; he had taken great pains to see everything, and his opinion was that to-day the Northampton Hospital was replete with everything conducive to the treatment of disease, replete with every facility modern science demanded, and complete in the sense that it provided accommodation capable of the very highest and best use. (Applause.) He thought it only right to say, as he had felt the responsibility of that matter, that the whole of the work of the new building and the conversion of the old reflected the greatest credit on all concerned. The architects, Mr. R. Greene and Mr. Dorman, and the builder, Mr. Henry Martin, had carried out their work well, and it was a remarkable fact that such buildings as they were now possessed of should be put up with such little difficulty. After a reference to the coolness and cleanliness of the wards, the speaker mentioned the good work which would be done at the hospital, and said it ought to command the support of working men. The result of the alterations was that patients even after a serious operation perfectly recovered and left the hospital within fourteen days. In London they considered about 19 days as the minimum, but the patients did not have the advantage of the splendid air which the patients had provided for them on the fine site on which the new hospital stood. (Applause.) After expressing the opinion that the direct result of the rebuilding of the hospital at a cost of £30,000 was that 30,000 working days each year would be added to the earning power of the artisan classes of the town and county of Northampton, the speaker suggested that the improvements made merited the greatly increased support of the working classes, both through the Hospital Week Movement and by means of regular weekly contributions.

Taking the hospital all round, it might be regarded as one of the best county hospitals in England. By its erection those who were responsible for it had done great service to the hospitals and to the medical profession of the country. They had succeeded in getting a perfected hospital; an incomparable building at a cost proportionately of about one-third of the amount that was being spent in the building of sanatoria for tubercular cases. Including everything, the scheme had been accomplished at a cost of £200 per bed, whereas ordinarily it was considered that between £500 and £600 had to be expended. It was certainly a matter for pride that they had in the circumstances which led to the erection of the building succeeded in getting a modern hospital replete in every department and thoroughly up-to-date, at something like one-third the cost which prevailed amongst kindred institutions throughout the country.

As they had modernised the building, so he hoped those responsible would modernise their system of management, and insist on having the highest development and efficiency of which the new hospital was capable. He suggested that they should consider the desirability of bringing the Finsen light and all the possibilities of the light treatment to the service of the poor. In the electrical department they had accommodation as good as anything in the world—certainly as good as Finsen himself had in Copenhagen, so that they might readily place themselves in a position not only to confer an inestimable benefit on the working classes, but they might afford facilities for the reception of patients who could afford to pay a fee. Nursing, too, was being everywhere developed. Every hospital which aimed at thoroughly training its nurses should give them an opportunity, if they desired to exercise it, of joining the private staff. If such staffs were everywhere instituted, everyone who wanted a fully-trained nurse whose character and knowledge were absolutely ascertainable and known, could have their wants satisfied by the simple means of addressing their applications to the nearest hospital. It was the wisest solution of a great difficulty, and it would bring grist to the mill. Other suggestions made by Sir Henry were those of providing literature for the use of the nurses, whom they expected to be highly intelligent women, and for the training of whose intellect they should certainly make provision. He also recommended that electric lifts should be provided.

In his concluding observations, Sir Henry, whilst acknowledging the good work accomplished by the medical and surgical staff, remarked that the erection of the new hospital was in a great degree due to two men—Lord Spencer and Mr. Adnitt. (Applause.) Throughout they had shown themselves most keenly anxious for the success of the movement to give Northampton a modern hospital. If any man deserved the gratitude of the townspeople it was Mr. Adnitt, who had given generously of his money and his energy, and who spared no pains to do it service. (Applause.)

Mr. H. Manfield in his reply referred to the great extent to which the hospital authorities were indebted to Sir Henry Burdett, who had not only given it the benefit of his extensive experience, but had also assisted materially in raising the necessary money. Alluding to the observations made with regard to the Finsen light, Mr. Manfield said all desired that the hospital should be second to none in the country, and so far as means would permit the Governors would do their utmost to ensure its being constantly up-to date. (Applause.)

Amongst the other speakers were Mr. Adkins, who paid a high tribute to Dr. Buszard; Mr. Perciyal, who expressed his satisfaction with the new building, and his belief that as a result of the improvement effected the time elapsing between operations and recovery had been curtailed by at least a half; the Mayor, in proposing the toast of the "Architect and Builders," stated that in his opinion the maximum of result had been obtained at the minimum of cost; Dr. Milligan, Dr. Hichens, Mr. Adnitt, and Mr. Bruce Cooper.

---

The foundation-stone of a new fever hospital has been laid at Peterhead. The cost of the institution is estimated at £4,000.

